# Interdomain Contacts Control Native State Switching of RfaH on a Dual-Funneled Landscape

**DOI:** 10.1371/journal.pcbi.1004379

**Published:** 2015-07-31

**Authors:** César A. Ramírez-Sarmiento, Jeffrey K. Noel, Sandro L. Valenzuela, Irina Artsimovitch

**Affiliations:** 1 Departamento de Biología, Facultad de Ciencias, Universidad de Chile, Ñuñoa, Santiago, Chile; 2 Center for Theoretical Biological Physics, Rice University, Houston, Texas, United States of America; 3 Department of Microbiology and The Center for RNA Biology, The Ohio State University, Columbus, Ohio, United States of America; Fudan University, CHINA

## Abstract

RfaH is a virulence factor from *Escherichia coli* whose C-terminal domain (CTD) undergoes a dramatic α-to-β conformational transformation. The CTD in its α-helical fold is stabilized by interactions with the N-terminal domain (NTD), masking an RNA polymerase binding site until a specific recruitment site is encountered. Domain dissociation is triggered upon binding to DNA, allowing the NTD to interact with RNA polymerase to facilitate transcription while the CTD refolds into the β-barrel conformation that interacts with the ribosome to activate translation. However, structural details of this transformation process in the context of the full protein remain to be elucidated. Here, we explore the mechanism of the α-to-β conformational transition of RfaH in the full-length protein using a dual-basin structure-based model. Our simulations capture several features described experimentally, such as the requirement of disruption of interdomain contacts to trigger the α-to-β transformation, confirms the roles of previously indicated residues E48 and R138, and suggests a new important role for F130, in the stability of the interdomain interaction. These native basins are connected through an intermediate state that builds up upon binding to the NTD and shares features from both folds, in agreement with previous *in silico* studies of the isolated CTD. We also examine the effect of RNA polymerase binding on the stabilization of the β fold. Our study shows that native-biased models are appropriate for interrogating the detailed mechanisms of structural rearrangements during the dramatic transformation process of RfaH.

## Author Summary

To carry out their biological functions, proteins must fold into defined three-dimensional structures. In most proteins, a single fold determined by the amino acid sequence, and sometimes influenced by environmental conditions, is believed to be suited for each protein’s dedicated task. However, some proteins challenge this broadly accepted paradigm, adopting different structures that can enable diverse roles or trigger pathological responses, such as prion diseases. *Escherichia coli* RfaH constitutes a dramatic example of this atypical behavior. RfaH C-terminal domain folds into either a helical bundle that binds to the N-terminal domain and inhibits unregulated recruitment to the transcription complex or, in the presence of a specific DNA target, into a stand-alone β-barrel structure that binds to the ribosome and couples transcription and translation of RfaH-dependent genes. To understand the mechanism of this structural rearrangement, we performed molecular dynamics using a model where the stabilizing interactions from both folds are integrated. Our results argue that this transformation requires destabilization of the domain interface, is favored by interactions between the N-terminal domain of RfaH and RNA polymerase, and proceeds via a bound intermediate state that connects both folds.

## Introduction

It has been more than 50 years since the protein-folding problem was first proposed [1]. Since then, several experimental [2,3] and theoretical approaches [4,5] have deepened our understanding of the energy landscape that guides a protein to its unique, thermodynamically-stable three-dimensional structure, the so-called native state, required to carry out its biological function [6]. However, the concept of the unique native state and the “one sequence/one fold” paradigm are challenged by transformer proteins [7] that are able to adopt multiple, highly-dissimilar but thermodynamically-stable configurations.

Several proteins capable of transforming into another native state in response to their cellular environment have been described, such as the ribosomal protein L20 from *Aquifex aeolicus*[8] and the human chemokine lymphotactin [9], the latter being extensively studied both experimentally [10] and computationally [11]. In both proteins the native state switching involves transitions between unrelated regions: the unfolding of one region of the protein is accompanied by folding of a different region. In other cases, such as the human mitotic spindle protein Mad2 [12], the structural transition involves conformational rearrangements where several secondary structure elements are maintained while the tertiary structure contacts are reorganized. Another example is the membrane-fusion homotrimer glycoprotein hemagglutinin from the influenza virus, where a metastable fold is created by cleaving a precursor protein, which, upon release by changes in pH, undergoes a large-scale secondary, tertiary and quaternary structural rearrangement crucial for delivering the viral contents into host cells [13,14].

**Fig 1:**
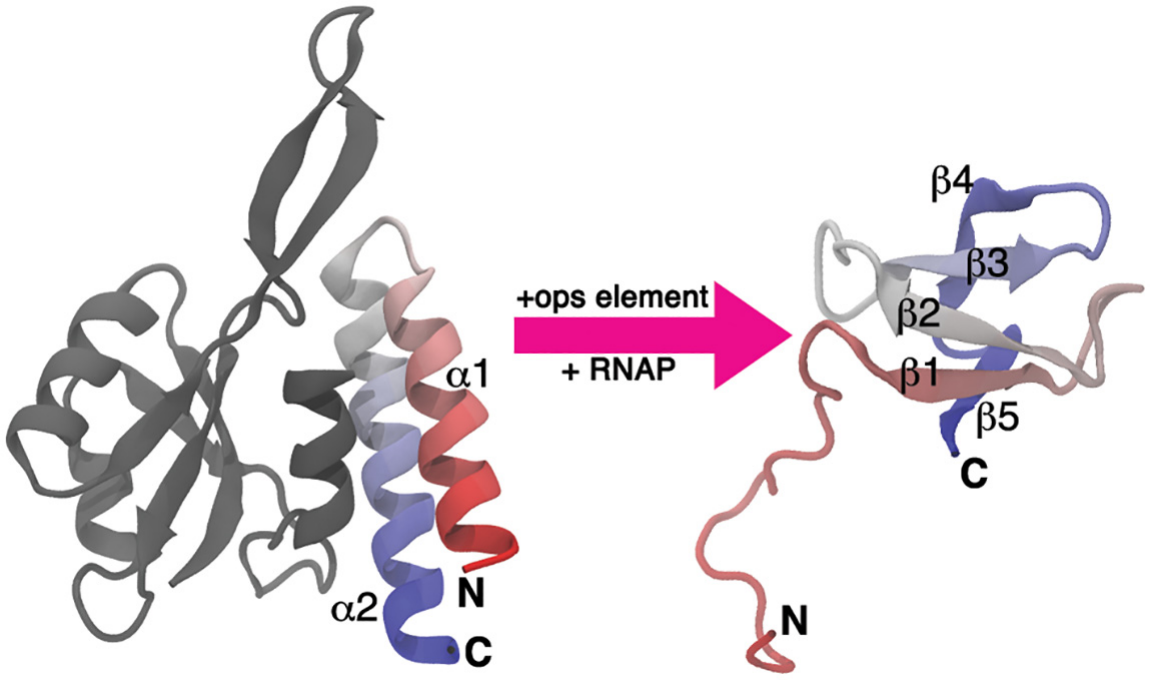
Structural transformation of the RfaH CTD. Domain dissociation is triggered upon binding of the NTD (gray) to its target *ops* (*operon polarity suppressor*) element DNA, relieving the autoinhibited state and allowing the transformation of the CTD (colored) from an α-helical hairpin (left) towards a five-stranded β-barrel (right). Note that the NTD and CTD are connected by a linker that does not order within the crystals and therefore is not shown in the figure. The accession codes for the structure of full RfaH in the α fold and of the excised CTD in the β fold are 2OUG and 2LCL, respectively.

Recently, an extreme case of a structural transformation has been described for the virulence regulator RfaH from *Escherichia coli*, which belongs to the NusG family of transcription elongation factors present in all three domains of life [15]. These proteins contain an α/β N-terminal domain (NTD) that binds to RNA polymerase (RNAp) and acts as a processivity clamp that locks around the transcribed DNA [15]. The NTD is connected through a flexible linker to the C-terminal domain (CTD) that in most NusG proteins is folded as a β-barrel [16]. In contrast, though still connected by a flexible linker, the CTD of RfaH folds as an α-helical hairpin that is stabilized into tight association with the NTD through interdomain interactions [17] (Fig 1). In this conformation, the CTD plays an autoinhibitory role by occluding the RNAp binding site of the NTD and preventing RfaH binding to the transcription complexes in the absence of a recruitment DNA signal.

Strikingly, NMR studies revealed that the isolated CTD folds into the five-stranded β-barrel structure seen in other NusG-like proteins (Fig 1)[18]. The ability of the CTD to refold from an α-helical hairpin into a β-barrel has been also evaluated in the context of the full protein by several approaches. First, destabilization of interdomain interactions through disruption of the salt bridge between residues E48 from the NTD and R138 from CTD allows coexistence of both folds at equimolar equilibrium [18]. Second, proteolytic cleavage of the flexible linker that connects both domains through an engineered TEV site wherein leads to refolding of the CTD into the β conformation [18]. Finally, domain swapping of the CTD and NTD does not affect the structure and function of RfaH, reinforcing the idea that interdomain contacts are the key factor determining the CTD fold [19]. These observations suggest that the CTD spontaneously refolds into a β-barrel upon domain dissociation (Fig 1), an event that is thought to be triggered when RfaH binds to its target *ops* (operon polarity suppressor) DNA [17]. In this scenario, domain dissociation enables the protein to bind to the *ops*-paused RNAp and permits the conformational transition of the CTD towards the β fold, which binds to the ribosomal protein S10 similarly to *E*. *coli* NusG [18]. Contacts with S10 are thought to enable the dramatic activation of RfaH-dependent operons by a combination of two mechanisms: recruitment of the ribosome to mRNA in lieu of a missing Shine-Dalgarno element [18] and subsequent coupling of transcription and translation that inhibits premature termination of RNA synthesis by Rho [20].

The dramatic conformational change of RfaH constitutes an intriguing problem by itself, since the folding mechanism underlying the structural rearrangements that occur during the transformation process is currently unknown. In addition, the detailed analysis of RfaH transformation will provide new insights about massive conformational changes towards alternative native or misfolded states that occur in other proteins. In this regard, computer simulations can provide important information about these conformational changes and at the same time overcome many of the difficulties that may arise while following these structural rearrangements experimentally. Studies of the structural transitions during the α-to-β conversion of the isolated CTD of RfaH using molecular dynamics with empirical force fields have been recently described [21,22], which hint at the presence of partially unfolded intermediates on the folding pathway. However, these simulations do not include the NTD of RfaH and thus neglect any involvement of the interdomain contacts shown to thermodynamically control the transformation process.

Inspired by this and by the fact that all the information required to determine the CTD fold is encoded by RfaH itself [19], we investigated the dramatic conformational change of the CTD of RfaH in the context of the full protein using structure-based models [23] that have been developed based on the energy landscape theory [24] and the principle of minimal frustration [4]. These models are biased towards the native state by the explicit inclusion of its topology into the energy Hamiltonian, such that all native interactions are stabilizing. The robustness of these models has been demonstrated by the reproduction of the observed folding and binding mechanism of several proteins [5,25], and their applications have been recently extended to the study of complex folding mechanisms by generalizing to multiple-basin energy landscapes [26–31]. Using these dual-basin structure-based models, we were able to follow the reversible interconversion between the α and β folds of the CTD of RfaH in the context of the full-length protein. Our results show that the structural transition between the folds is connected through an obligate intermediate, and that weakening of the interdomain contacts is sufficient to trigger the interconversion. The structural features of the intermediate states described herein are consistent with local frustration and secondary structure propensity analysis of the CTD. Moreover, our model allowed us to define the interdomain residues that are most responsible for controlling folding-upon-binding of the CTD into the α state. These results are in excellent agreement with the current experimental evidence of the dramatic conformational transition of RfaH and provide new insights into its mechanism.

## Results and Discussion

### Dual-basin energy landscape of RfaH provides a description consistent with experimental data

The folding of proteins is typically well described by structure-based models because a protein’s funneled energy landscape is selected to be consistent with the structure of the native state [4,32]. In the case of RfaH, the structure of its CTD has been solved either in the context of the full protein by X-ray crystallography [17] or in isolation by NMR [18], showing striking structural differences. In the full protein, the folded state of the CTD corresponds to an α-helical hairpin that establishes extensive contacts with the NTD [17]. However, the isolated CTD folds into a five-stranded β-barrel [18] observed in the homologous NusG-like transcription factors from bacteria, archaea and eukaryotes [33]. Both folded states represent low free energy ensembles that the same sequence can fold into. Therefore, in RfaH, evolution has selected a sequence that is consistent with two structures, which can be represented with a *dual-basin* structure-based model. In this case, the enthalpy contributions from both folds are combined such that both structures of the CTD are explicit energy minima. This dual-basin approach has been previously used to study the competing formation of symmetry-related native and mirror structures of Rop dimer [26,34,35] and the B domain of protein A [36] and the large-scale structural rearrangement of the human chemokine lymphotactin [29] and the influenza virus glycoprotein hemagglutinin [14].

**Fig 2:**
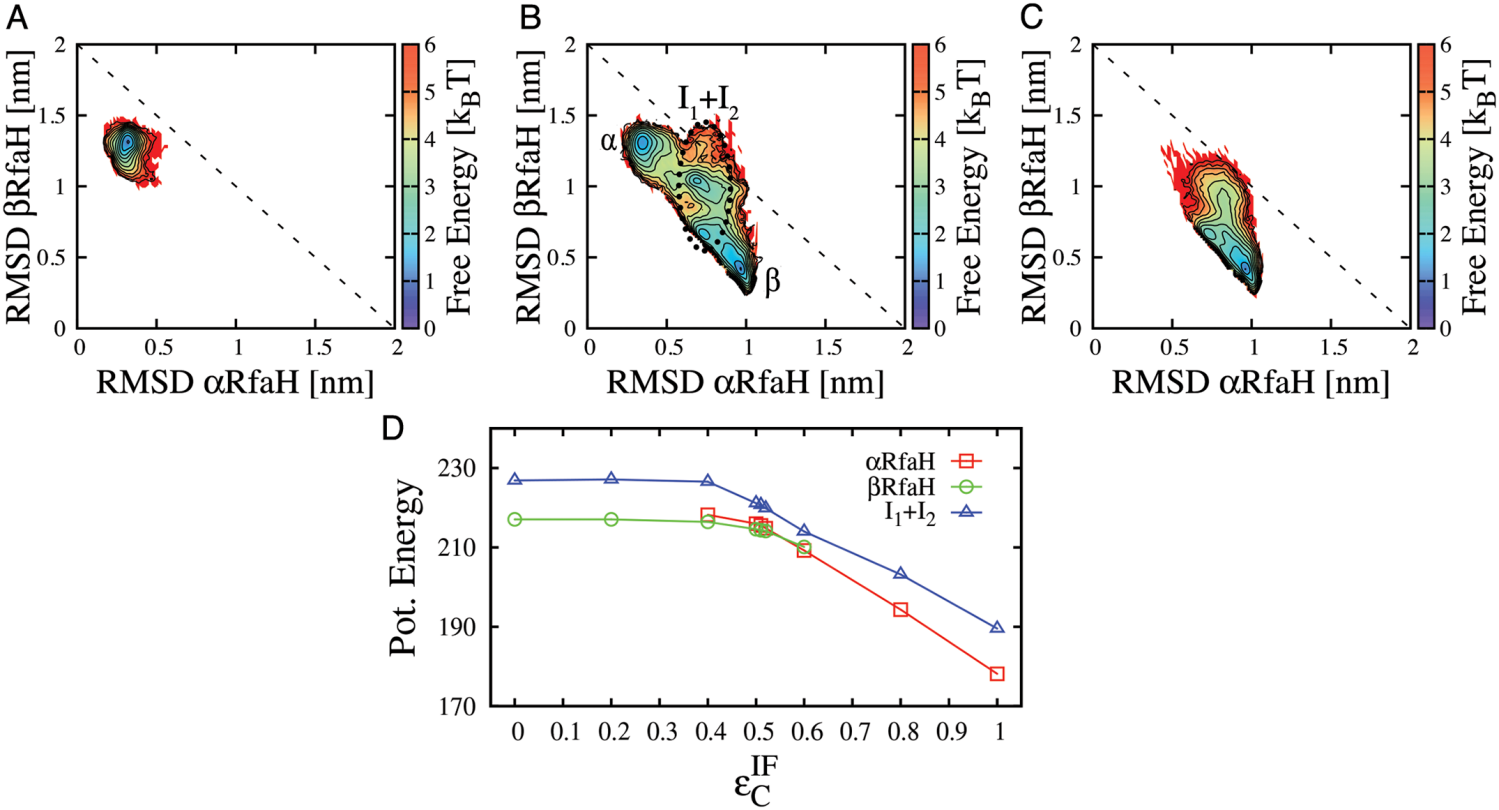
Coexistence of the α and β folds of RfaH can be tuned by changing the strength of interface contacts. Contour plots show 2D free energy profiles obtained from simulations of the full RfaH protein. The strength of the interdomain contacts $\varepsilon_{\text{C}}^{\text{IF}}$ was equal to intradomain contacts (A), reduced in ~50% (B) or deleted (C). The reaction coordinates are RMSD of the CTD (residues 115–162) to either the all-α or the all-β crystal structures. The contour lines define steps of 0.5 *k*_B_T* and the temperature is 0.92 T_F_^β^. Interconversion between α and β goes through obligate intermediate states, indicated through a black dotted ellipse (B). The potential energy for the α, β, and intermediate ensembles is plotted in (D). While both the α and β configurations can form interface contacts, α is able to form more and becomes relatively more energetically stable at high $\varepsilon_{\text{C}}^{\text{IF}}$. α is rarely sampled at $\varepsilon_{\text{C}}^{\text{IF}}<0.4$ and β is rarely sampled at $\varepsilon_{\text{C}}^{\text{IF}}>0.6$. Despite its higher enthalpy, the disordered intermediate ensemble is sampled because of its larger entropy.

The thermodynamics of the dual-basin model of RfaH is consistent with experimental findings (Fig 2). First, when connected to the NTD, the thermodynamic minimum of the CTD is the α fold [17] (Fig 2A, $\varepsilon_{\text{C}}^{\text{IF}}=\varepsilon$). Second, when interaction with the NTD is removed, the CTD folds into β (Fig 2C, $\varepsilon_{\text{C}}^{\text{IF}}=0$). The β-fold is observed when the CTD is excised from the full RfaH protein by proteolytic cleavage of the interdomain linker [18]. Finally, there exists an interface stability that allows for coexistence between the folds (Fig 2B and S1 Fig). Experimentally, both folds were detected when destabilizing mutations such as the NTD substitution E48S were introduced into the interface between the CTD in the α fold and the NTD [18]. In the simulation, if the overall affinity between the NTD and CTD is reduced by uniformly lowering the strength of the interface contacts by ~50%, α and β are equally probable and exhibit transitions between the states.

The important role of the interface contacts in determining the fate of the CTD is readily understood by considering the differences in structure between the α and β folds. The α-helical CTD forms a large interface with NTD, while the β-barrel buries many of the interface residues involved in these contacts. Therefore, decreasing the strength of these contacts destabilizes α more than β (Fig 2D). Landscapes for additional intermediate levels of $\varepsilon_{\text{C}}^{\text{IF}}$ are shown in S2 Fig. At all levels of interface contact strength, a subset of CTD lies in intermediate configurations. In the next section we discuss the role of these intermediates in the folding route connecting α and β.

### The α and β folds of the CTD of RfaH interconvert through an intermediate ensemble that is bound to the NTD

Although the wild-type RfaH is only known to exist with the CTD bound in the α fold in the absence of the transcription elongation complex (TEC), the use of domain-swapped [19] and single-residue [18] mutants provided strong experimental evidence of interconversion between the α and β folds of the CTD in the context of the full-length protein. Domain swapping suggested that the protein can fold back into the α fold even when the CTD is the first element to be translated [19]. The NTD substitution E48S destabilizes the interface such that the CTD coexists in both folds at equimolar equilibrium [18]. Therefore, a model where the strength of the interface contacts is tuned so that both CTD folds are equally probable, as when $\varepsilon_{\text{C}}^{\text{IF}}=0.51\varepsilon$, is not only useful for describing the interconversion pathway for the wild-type protein but also describes protein models that are experimentally realizable.

The energy landscape presented for RfaH when $\varepsilon_{\text{C}}^{\text{IF}}=0.51\varepsilon$ shows that its native basins are connected through obligate intermediate configurations (Fig 2B). As a control, to verify that the intermediate ensembles are not an artifact caused by our choice of dihedral mixing, we also performed simulations using a dual-basin dihedral potential as described elsewhere [14]. This potential further stabilizes the intermediate (S3 Fig).

**Fig 3:**
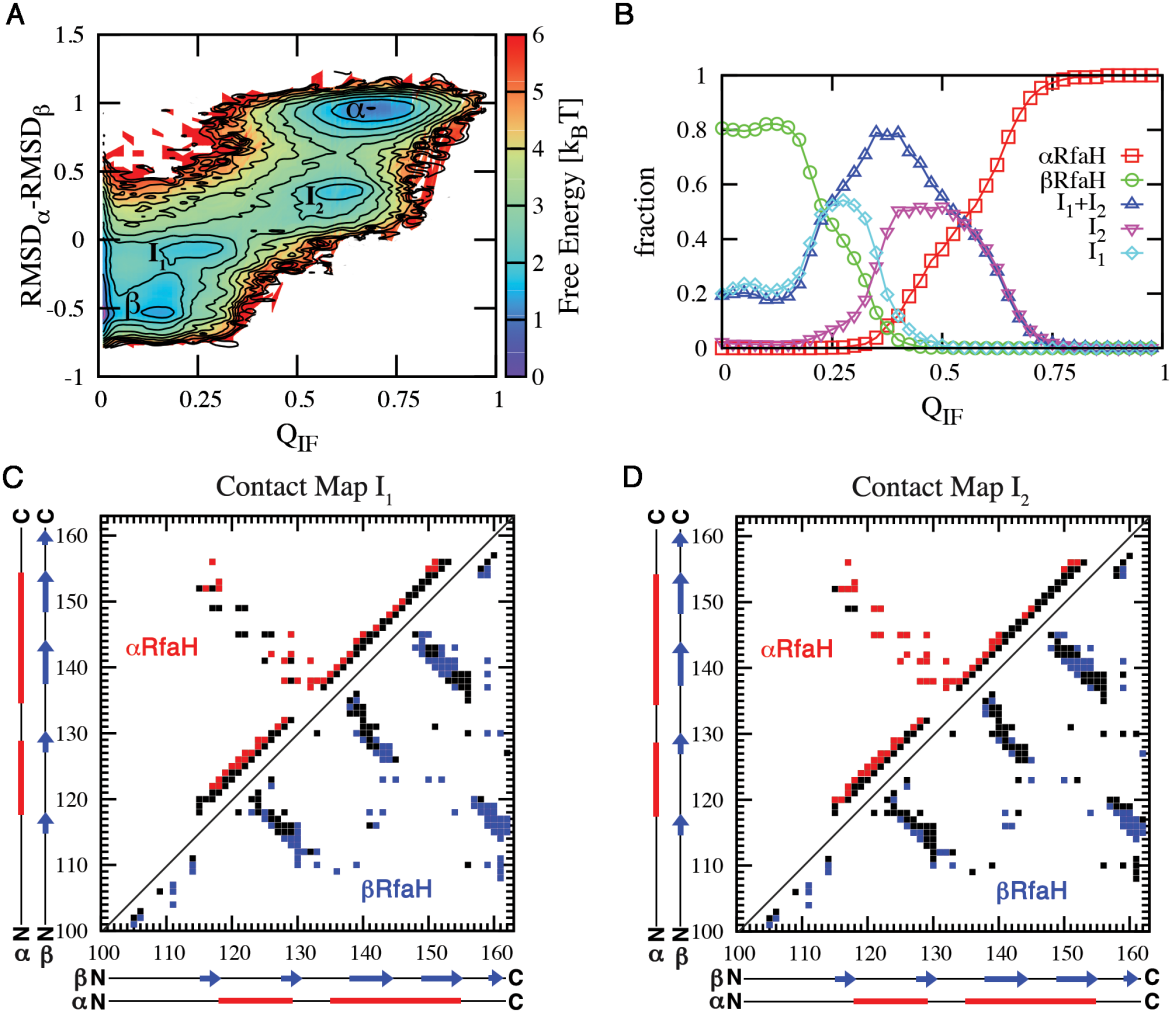
Interconversion between α and β involves folding intermediates that are interacting with the NTD. (A) Free energy landscape at $\varepsilon_{\text{C}}^{\text{IF}}=0.51\varepsilon$, where the probability of α and β are equal, shows a three-state folding landscape: β/I_1_ <-> I_2_ <-> α. (B) Comparing ensembles as a function of Q_IF_, the fraction of interface contacts formed, shows that β and I_1_ can form without interacting with the NTD, whereas I_2_ and α are only found when interacting with NTD. The populations of β and I_1_ near Q_IF_ ~ 0.2 indicates that these configurations present a small amount of surface residues that make contacts with the NTD in the α structure. States were defined as RMSDβ–RMSDα ranges: [-0.80, -0.30] as the β state, [0.80, 1.20] as the α state, and [-0.25, 0.00] and [0.15, 0.60] as the I_1_ and I_2_ ensembles. Contact maps of intermediate states I_1_ (C) and I_2_ (D) were constructed by isolating these ensembles from simulations at T = 0.92 T_F_^β^ and then determining the native contacts from αCTD (red) and βCTD (blue) that have a contact probability > 0.5.

The transformation process takes place in the context of the full-length protein and involves interactions between the domains. The fraction of interface contacts Q_IF_ quantifies the level of interaction between NTD and CTD, while an RMSD difference, RMSDβ–RMSDα, measures the structural state of the CTD (Fig 3). The free energy landscape along these coordinates shows that β and α are connected through two intermediate states, I_1_ and I_2_, and that these intermediates are forming contacts with the NTD (Fig 3A). β and I_1_ are populated both at Q_IF_ = 0 and Q_IF_ > 0, while I_2_ and α are only populated when interacting with the NTD (Fig 3B). α is fully populated when the fraction of interdomain contacts exceeds 75% (Fig 3B). Hence, our data suggests a three-state folding process β/I_1_ <-> I_2_ <-> α, where the interconversion between β/I_1_ and I_2_ occurs while interacting with the NTD.

To verify the kinetic relevance of our projection of the free energy landscape in Fig 3, we performed a long constant temperature simulation and counted the transitions between the different ensembles (S4 Fig). Transitions only occur between states α <–> I_2_, I_1_ <-> I_2_ and β <-> I_1_, with the latter being most frequent, in line with the low free energy barrier separating these ensembles. These transitions are consistent with the three-state folding process previously defined. Additionally, the unfolded state is not sampled whatsoever in these simulations.

Recent simulations using implicit and explicit solvent force fields have suggested that the isolated CTD traverses an intermediate during kinetic simulations of the one-way α-to-β transformations [21,22]. This result is consistent with the β <-> I_1_ dynamics that can transition without interacting with NTD. Finally, it is worth noting that the presented folding landscape for RfaH differs from other transformer proteins such as lymphotactin, where stepping into the unfolded state is required [10].

### Features of the intermediates connecting the α and β states of RfaH

The intermediates emerge as low free energy combinations of native contacts contributed by the two input contact maps for RfaH. To structurally describe the intermediate ensembles we determined which native contacts are formed (Fig 3). A native interaction is considered formed in these ensembles if their contact probability is greater than 0.5.

I_1_ is most similar to β (Fig 3C). Most of the interactions between strands β3-β4, β1-β5 and a large portion of the contacts between strands β1-β2 and β2-β3 are established (Fig 3C). This is similar to previous depictions of the α-to-β conversion of the isolated CTD using Markov state models, where strands β2, β3 and β4 are thought to be formed earlier during the transition towards the β state [22].

I_2_ is most similar to α, having most of the interactions between strands β1-β2 and β2-β3 unformed (Fig 3D). Almost all of the non-local interactions between helix α1 and α2 are formed, but there is still partial unwinding of helix α2, while α1 seems to be stable. A higher probability of local contacts in helix α1 differs from molecular dynamics simulations of the isolated CTD, which suggested that this element is less stable [21,22]. However, this discrepancy would be expected as I_2_ forms extensive interactions with the NTD that can modify its stability.

To gain insight into the intermediate ensembles predicted by the dual-funneled structure-based model, we estimated the local frustration of the α and β folds and the secondary structure propensity of the sequence of RfaH CTD using the protein frustratometer [37] and Jpred-3 [38] webservers, respectively. Local frustration analysis shows that most of the interactions that support robust folding (i.e. minimally frustrated contacts) of the α-helical state of RfaH CTD correspond to the non-local interactions between helix α1 and α2, most of the local interactions of helix α1 and local interactions between residues 139–146 of helix α2 (S5 Fig). Interestingly, the C-terminal end of helix α2 suggests that this region is highly frustrated (S5 Fig). Thus, there is consistent evidence from both conformational entropy (folding simulations using structure-based models) and native state heterogeneity (frustration) for the structure of the intermediate I_2_ (Fig 3D). The β fold is highly consistent, only having a small amount of frustration localized in interactions between strands β2–β3 and β3–β4 (S5 Fig). These features of the β fold are also consistent with the overall structure of the intermediate I_1_ (Fig 3C). Lastly, secondary structure prediction based on the sequence of RfaH CTD suggest that residues 136–145 have some helical propensity (S5 Fig), thus being consistent with the presence of helical local interactions that are featured by this region in the intermediate state I_1_ (Fig 3C). In line with our results, recent secondary structure prediction analysis of RfaH CTD [39] showed that residues 141–145 encompass a Leucine-rich region (sequence LLLNL), which has a high propensity to adopt helical configurations, whereas the homolog region in NusG is mainly composed by valine and isoleucine, which are known to favor β structures [40,41]. The same fragment is present in several unrelated structures solved in the Protein Data Bank and also exhibit an helical structure [39], strengthening the idea that the sequence of RfaH CTD has some localized α-helical propensity and that this sequence motif can be used to identify other transformer proteins along the evolution of the NusG family.

**Fig 4:**
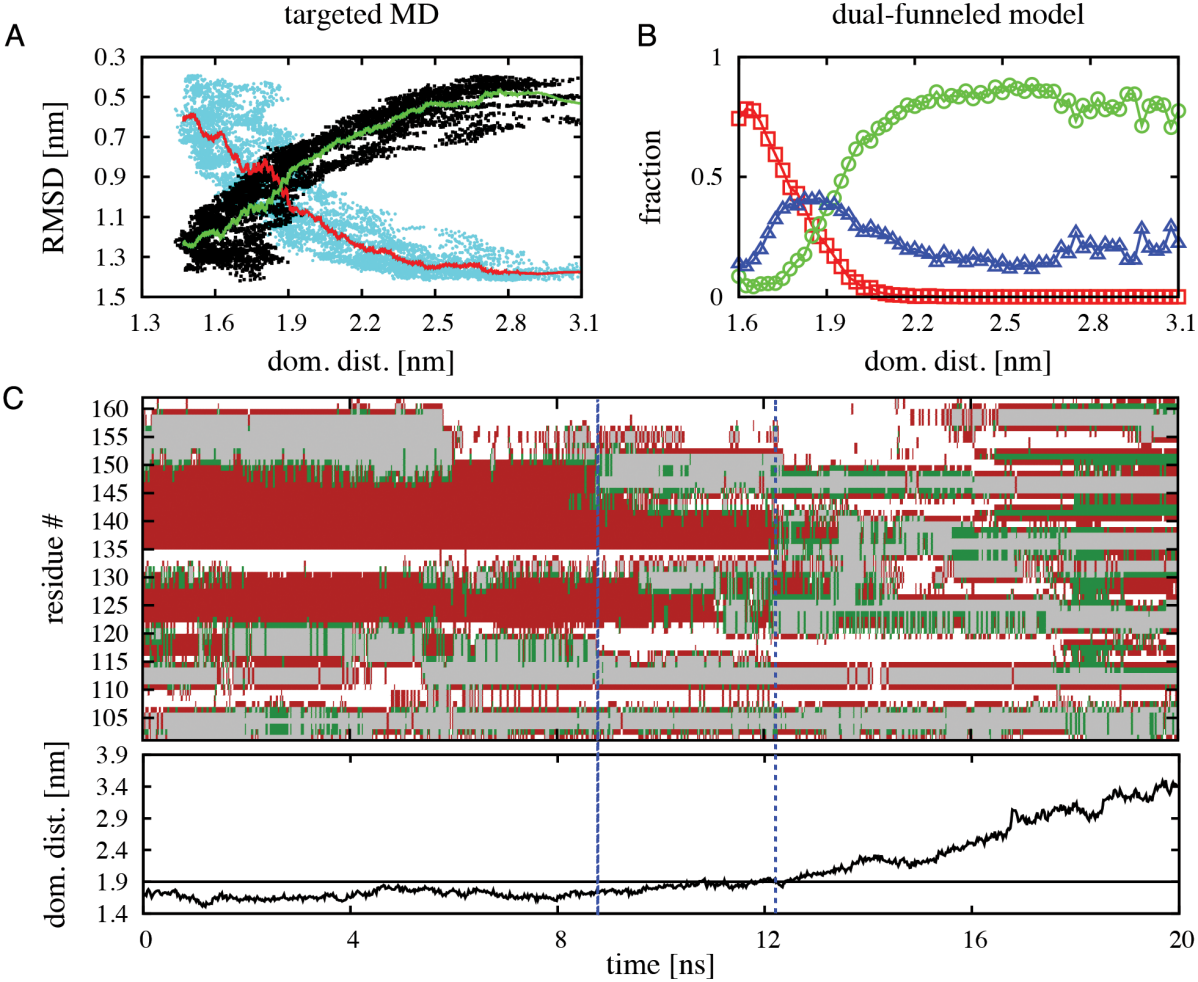
TMD simulations of the α-to-β structural transformation of full RfaH protein in explicit solvent. (A) Change in RMSD of αCTD (cyan dots) and βCTD (black dots) for all TMD simulations as a function of the center of mass distance between the NTD and CTD domains. The red and green lines indicate the average change in RMSD of αCTD and βCTD, respectively. (B) Fraction of CTD in the α (red symbols), intermediate (blue symbols) and β (green symbols) folds as a function of the center of mass distance between the NTD and CTD domains estimated from dual-funneled simulations at $\varepsilon_{\text{C}}^{\text{IF}}=0.51\varepsilon$. (C) Typical TMD trajectory of the change in secondary structure content per CTD residue as a function of time. The color code corresponds to helices in red, extended structures in green and turns in grey, while white regions represent coils. The dashed blue lines indicate when significant loss of helical content in the C-terminus of helix α2 occurs. The plot on the bottom indicates the change in the center of mass distance between the NTD and CTD during the simulation, where the black line at 1.9 nm indicates when the dissociation process occurs.

To further validate the structural features of the intermediate states predicted by our dual-funneled model during native state switching of RfaH CTD, we performed targeted molecular dynamics (TMD) [42] of the α-to-β transformation of the full RfaH protein in explicit solvent. It is worth noting that the reaction coordinate that steers RfaH towards the β fold in TMD is defined through the RMSD to the target structure, namely the βCTD, and hence there is no direct perturbation of the NTD-CTD interface interactions. By use of a steering force of 672 kcal·mol^-1^·Å^-2^ over the 62 Cα atoms of the CTD, we collected 7 TMD simulations that each successfully reached a β-like fold, as indicated by measurement of the RMSD against the CTD in the β fold (on average ~0.5 nm), totaling 140 ns of simulation. As seen in Fig 4A, the α-to-β transition is accompanied by an increase in distance between the CTD and NTD domains, in a similar fashion as the increase in the fraction of CTD in the β fold at increasing domain distances observed in our dual-funneled models (Fig 4B). As illustrated in Fig 4C, our α-to-β TMD simulations show that a significant loss of helical structure is observed in helix α2 (residues 142–151) between 8 ns and 12 ns of simulation, before dissociation of the CTD and NTD domains occur. This observation is fully compatible with the structural features of the I_2_ intermediate described using dual-funneled models (Fig 3D) and with the local frustration analysis of the CTD in the α fold (S5 Fig). After dissociation, the CTD accumulates extended secondary structure content related to formation of β-strands, although some helical content is still present, thus being compatible with the I_1_ intermediate previously described (Fig 3C).

Overall, our results provide good evidence that the transformation mechanism of CTD involves intermediate states that share structural features from both folds and that this process is not simply related to its topology, but a combination of the dual basin of CTD and its interface interactions with NTD.

### Role of interface contacts in the large structural change of RfaH

The transformation event triggered by binding of RfaH to the *ops*-paused RNAp [17] is likely related to allosteric communication between the NTD *ops* binding site to the NTD-CTD interface. Thus, understanding how the interface is involved in the transformation between α and β is crucial for understanding the activity of RfaH. Both the β and I_1_ states can be populated in the absence of interface interactions (Fig 3B). Hence, the key binding step allowing the structural change corresponds to the β/I_1_ <-> I_2_ transition, since the I_2_ <-> α occurs with the CTD already bound to the NTD. Therefore, we calculated the contact probability of each interdomain contact in the transition state ensemble (TSE) of this folding step to determine the residues responsible for binding between the NTD and CTD during the conformational change and enabling RfaH to act as a sequence specific regulator of gene expression.

**Fig 5:**
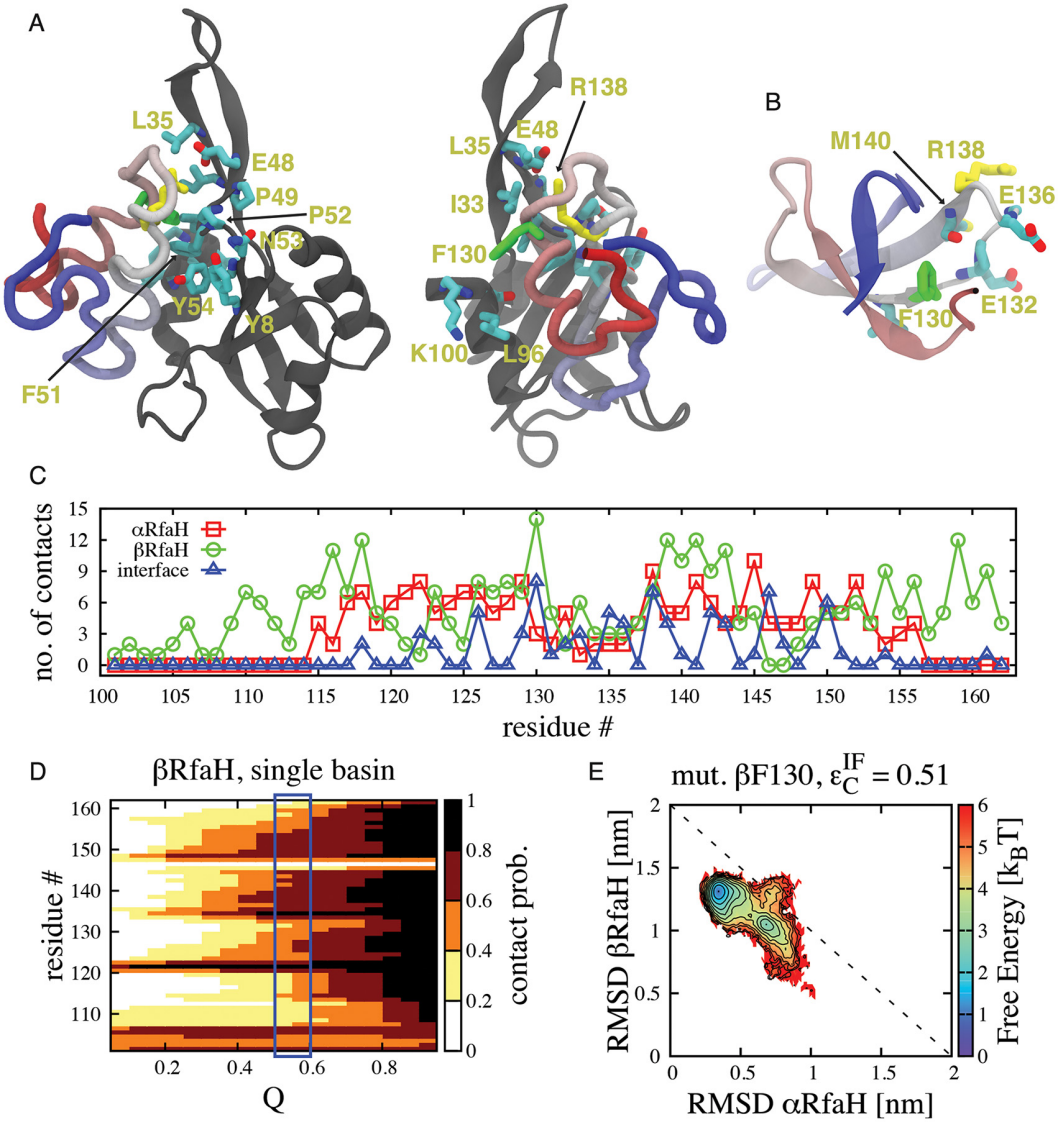
Structural features of the binding TSE that connects the native conformations of RfaH-CTD in dual-basin structure-based models. (A) Stick representation of residues involved in interface contacts between the NTD (gray) and CTD (colored) at the transition state separating I_1_ and I_2_ that possess a contact probability > 0.5. Of all residues, the most important ones are F130 (green), which forms part of the hydrophobic core of CTD in the β fold (B), and R138 (yellow), which interacts with E48 forming a salt bridge and is exposed on the surface of the CTD in both the α and β folds. (C) Number of native contacts per CTD residue in the α and β folds and also interdomain native contacts in the α fold, highlighting the dual role of residue F130 in stabilizing both states. (D) The contact probability per residue as a function of the folding reaction coordinate Q is shown for βCTD. Its TSE (blue rectangle) is composed by residues that have a contact probability higher than 0.6. (E) Free energy landscape at $\varepsilon_{\text{C}}^{\text{IF}}=0.51\varepsilon$ upon deletion of contacts formed by residue F130 in the β fold, showing that its destabilization leads to favoring the α fold.

All of the residues that are key for the binding TSE of the I_1_ <-> I_2_ step (i.e. their contact probability is greater than 0.5) are located in the vicinity of residues E48 and R138 from the NTD and CTD, respectively. In fact, most of the residues that form the β-hairpin of the NTD (residues 30–52) are involved in binding of the CTD during this folding step (Fig 5A). Moreover, residue E48, whose substitution by serine allows experimental observation of the α and β folds of RfaH in 1:1 equilibrium [18], has a contact probability (averaged over all contacts where this residue is involved) of ~0.87, being one of the highest probabilities among all of the NTD interface residues.

In the TSE, the NTD interacts with residues I129, F130, E132, P133, G135, E136, R138 and S139 from the CTD, which are located in the loop connecting helices α1 and α2 and in the first turn of helix α2. Remarkably, most of the side chains of these interface residues (I129, E132, E136, R138) are pointing towards the surface in the β fold, therefore being readily available to interact with the NTD (Fig 5B). This architecture allows β/I_1_ to interact with the NTD without unfolding the hydrophobic core, significantly lowering the overall barrier to transformation.

The only interface residue involved in the binding TSE that also forms extensive hydrophobic contacts in β is F130, having the highest number of native contacts per residue in the β fold (Fig 5C), and unfolding it likely creates the small barrier separating β/I_1_ and I_2_. We tested the importance of F130 on the stability of the CTD in the β fold by first defining the TSE of this fold using single-basin models. Our simulations show that residues 105–107, 121–123, 134–143 and 148–154 have a contact probability in the TSE higher than 0.6 and define the folding nucleus (Fig 5D). It is important to note that these regions describing the folding nucleus of βCTD are also the firsts to exhibit β-strand formation in our TMD simulations (Fig 4C). Residue F130 has a slightly lesser contribution to the structure of the TSE by having a contact probability of ~0.5 in the single-funneled model. We then performed an *in silico* mutation of F130 through deletion of all the native contacts that this residue establishes in the β fold (named *βF130*) and performed simulations at $\varepsilon_{\text{C}}^{\text{IF}}=0.51\varepsilon$, thus testing the role of residue F130 on the stability of the β fold and the structural transformation of RfaH CTD. As shown by the contour plot in Fig 5E, removal of these contacts destabilizes the β fold and the intermediates, relative to the α fold of RfaH CTD. Altogether, these results highlight the dual role of F130 in stabilizing the hydrophobic core of the β fold and interacting with the NTD to stabilize the intermediates.

### β fold of RfaH-CTD is favored when RNAp is bound

RfaH is recruited to RNAp paused at the *ops* site [20]. While the details of how RNAp and *ops* initially induce the dissociation of the α fold CTD are not known, we show that having RNAp bound to RfaH is sufficient to maintain the CTD in its β fold. This is important since RfaH’s function of coupling transcription and translation requires the β-folded CTD to interact with the ribosomal protein S10.

**Fig 6:**
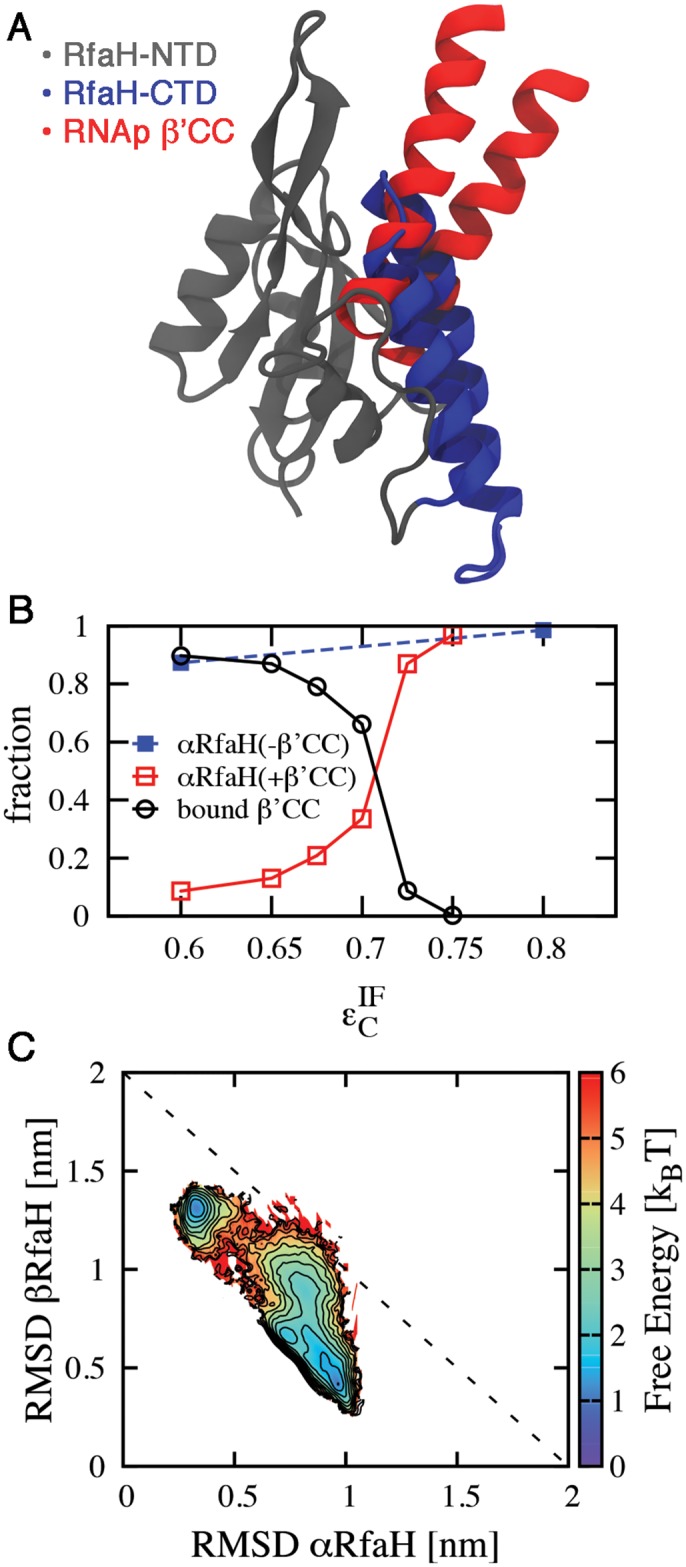
Effect of RNAp binding to the NTD in the folding ensemble of CTD. (A) Structural superimposition of the NTD of the full RfaH protein (gray) and *P*. *furiosus* Spt5 bound to RNAp (accession code 3QQC), showing that the CTD in the α fold (blue) occludes the binding site for the β’CC of *E*. *coli* RNAp, equivalent to the subunit A’ coiled-coil of *P*. *furiosus* RNAp (red). (B) Competitive binding of RNAp β’CC to the NTD leads to destabilization of the α fold of the CTD at higher $\varepsilon_{\text{C}}^{\text{IF}}$ values than in the absence of RNAp and is encouraged by reducing the strength of the interdomain interactions. (C) Folding thermodynamics at $\varepsilon_{\text{C}}^{\text{IF}}=0.675\varepsilon$ in the presence of RNAp β’CC show that its binding to the NTD leads to destabilization of the α fold and stabilization of the β fold.

Interface contacts occluded by RNAp binding were identified by superimposing the NTD of RfaH with its archaeal homologue Spt5 from *Pyrococcus furiosus*, which forms a heterodimer with Spt4 and is bound to the RNAp clamp domain (accession code 3QQC, Fig 6A)[43]. In this structure, residues 237–280 of the A’ subunit of *P*. *furiosus* RNAp form a coiled-coil equivalent to the β’CC of *E*. *coli* RNAp [43]. Residues 255–265 located on the tip of the coiled-coil structure interact with Spt5 and are equivalent to residues 282–292 of *E*. *coli* RNAp β’CC, whose replacement by a glycine linker completely disrupts the interaction between RfaH and RNAp [17].

In the resulting superimposition 53 out of 80 interface contacts are occluded, mostly in the vicinity of residue E48 (Fig 6A). Removal of these contacts from the dual-basin structure-based model mimics the effect of RNAp binding to the NTD, and leads to a strong destabilization of α (S6 Fig). At T = 0.92 T_F_^β^ and $\varepsilon_{\text{C}}^{\text{IF}}=\varepsilon$, the populations in β and intermediate states are 76% and 21% respectively, with only 1% of the CTD in the α-helical fold.

Since binding of β’CC does not actually remove the affinity of the CTD for NTD, the interaction with β’CC is actually a biomolecular process where β’CC and CTD compete for the NTD. We performed simulations where the β’CC of RNAp was explicitly included. As illustrated in Fig 6B, β’CC competes with RfaH CTD to bind to the NTD when $\varepsilon_{\text{C}}^{\text{IF}}\leq 0.75\varepsilon$, and effectively displaces the CTD when $\varepsilon_{\text{C}}^{\text{IF}}\leq 0.60\varepsilon$. Naturally, β’CC binding destabilizes the α fold of CTD by occluding its NTD interface (Fig 6C). Interestingly, the presence of β’CC raises the interface contact strength of the equilibrium between α and β from $\varepsilon_{\text{C}}^{\text{IF}}=0.51\varepsilon$ to $\varepsilon_{\text{C}}^{\text{IF}}=0.71\varepsilon$. If native RfaH has an equilibrium value of $\varepsilon_{\text{C}}^{\text{IF}}>\varepsilon$ (since α is dominant in the NMR structure), this is consistent with the fact that RNAp alone does not bind RfaH. Presumably, inclusion of the full RNAp with *ops* binding site would push the $\varepsilon_{\text{C}}^{\text{IF}}$ midpoint sufficiently above ε in order to shift the equilibrium towards bound β’CC and βCTD. Unfortunately a structure including these interactions is not yet available. These results can be sufficient to explain how RNAp is able to exclude the CTD from binding and favor its β fold.

### Concluding remarks

The complex α-to-β structural conversion of RfaH-CTD in the context of the full protein can be addressed using dual-basin structure-based models that integrate the topology of both native states into a single Hamiltonian. Our model is able to reproduce several features of this process that have been experimentally demonstrated or suggested from detailed molecular simulations, such as i) the disruption of interdomain interactions enables the coexistence of α and β; ii) the large structural change of RfaH as a three-state folding process β/I_1_ <-> I_2_ <-> α. Our results also give new insights about how this folding mechanism is coupled with NTD-CTD binding, the structural features of the intermediate ensembles and the key interdomain residues that permit binding during the β-to-α transformation of the CTD. Moreover, we propose that residue F130, which stabilizes several interactions with the hydrophobic core of βCTD and is exposed towards the interdomain interface in the α fold, is key to control the stability of the β fold and the TSE that separates both native basins. Overall, we find that in the presence of RfaH-NTD, the transformation mechanism of CTD is not simply related to its topology, but a combination of the dual basin of CTD and its interface interactions with NTD.

While most of these results arise from a structure-based model where the strength of the interfacial contacts has been homogeneously tuned to equally populate both folds, we also address a plausible scenario for the specific effect of RNAp after binding to the NTD. Once interactions of *ops* with its binding site in RfaH have allosterically triggered domain dissociation and allowed RNAp to bind to the newly exposed NTD surface (equivalent to reducing the strength of interdomain contacts below 0.75ε), steric hindrance of the formation of specific interdomain contacts by the RNAp β’CC favors the β fold of RfaH.

While our models overcome many of the challenges that can be found experimentally, the obtained results offer valuable starting points to guide *in vitro* experiments, such as mutational analysis of the NTD residues predicted to contribute for binding of the CTD and kinetic measurements of mutants of the F130 residue that would either lower the free energy barrier limiting the α-to-β conformational change or destabilize the β fold and favor the inactive state of RfaH, in order to gain a better understanding of the dramatic transformation of the CTD of RfaH.

## Methods

### Dual-basin structure-based models

Our simulations were performed using a coarse-grained structure-based model [5] generated using the SMOG server [44], where each residue is represented by a single bead centered at the coordinates of its corresponding Cα atom.

$$\begin{array}{c} V^{sb}=\sum_{\text{bonds}}\varepsilon_{r}{\left(r-r_{0}\right)}^{2}+\sum_{\text{angles}}\varepsilon_{\theta }{\left(\theta -\theta_{0}\right)}^{2}+\sum_{\text{dihedrals}}\varepsilon_{\phi }F_{D}\left(\phi -\phi_{0}\right)\\ +V_{\text{contacts}}+\sum_{ij\;\notin \text{ contacts}}\varepsilon_{\text{NC}}{\left(\frac{\sigma_{ij}}{r_{ij}}\right)}^{12}\\ F_{D}\left(\phi \right)=\left[1-\text{cos}\left(\phi \right)\right]+\frac{1}{2}\left[1-\text{cos}\left(3\phi \right)\right] \end{array}$$ (1)

In this model, bonds, angles and dihedrals are maintained by harmonic restraints, and non-bonded residues in contact in the native state are given attractive interaction while all other non-local interactions are treated as repulsive, as described in ref. 5. The terms *r*_0_, *θ*_0_, *ϕ*_0_ correspond to the values of bonds, angles and dihedrals in the native structure. The parameters ε_*r*_ = 100ε, ε_*θ*_ = 20ε, ε_*ϕ*_ = ε, ε_NC_ = ε weight the strength of each type of interaction. The functional form of the contact potential is:

$$V_{\text{contacts}}^{sb}=\sum_{ij\;\in \text{ contacts}}\varepsilon_{\text{C}}\left[5{\left(\frac{\sigma_{ij}^{0}}{r_{ij}}\right)}^{12}-6{\left(\frac{\sigma_{ij}^{0}}{r_{ij}}\right)}^{10}\right]$$ (2)

Where $\sigma_{ij}^{0}$ is the distance between the residue pair *i*,*j* Cα atoms in the native state and ε_C_ is the energy of the native contact.

The native contact maps for the full RfaH protein with its CTD in the α fold and for the CTD in the β fold were determined from structures deposited in the Protein Data Bank [45] with accession codes 2OUG and 2LCL, respectively (Fig 1). Loop residues 101–114 not solved in the crystal structure of the full RfaH protein were modeled using MODELLER [46] and were given no native contacts in the α fold. This approach is justified because small deletions, insertions, and substitutions in this loop do not affect RfaH function and thus presumable folding (IA, unpublished). The native contact map between residues separated in sequence by at least two amino acids (*i > j* + 2) was determined from each structure using the shadow map algorithm [47]. In order to account for the α-to-β conformational transition of the CTD of RfaH, the native contact potentials determined for both folds were combined as in Sutto et al [29]:

$$\begin{array}{r} V_{\text{contacts}}^{db}=\sum_{\begin{array}{c} ij\;\in \;\alpha \text{ non-interface}\\ \text{contacts} \end{array}}\varepsilon_{\text{C}}\left[5{\left(\frac{\sigma_{ij}^{\alpha }}{r_{ij}}\right)}^{12}-6{\left(\frac{\sigma_{ij}^{\alpha }}{r_{ij}}\right)}^{10}\right]\\ +\sum_{\begin{array}{c} ij\;\in \;\beta \\ \text{contacts} \end{array}}\varepsilon_{\text{C}}\left[5{\left(\frac{\sigma_{ij}^{\beta }}{r_{ij}}\right)}^{12}-6{\left(\frac{\sigma_{ij}^{\beta }}{r_{ij}}\right)}^{10}\right]\\ +\sum_{\begin{array}{c} ij\;\in \;\alpha \text{ interface}\\ \text{contacts} \end{array}}\varepsilon_{\text{C}}^{\text{IF}}\left[5{\left(\frac{\sigma_{ij}^{\alpha }}{r_{ij}}\right)}^{12}-6{\left(\frac{\sigma_{ij}^{\alpha }}{r_{ij}}\right)}^{10}\right] \end{array}$$ (3)

Where $\sigma_{ij}^{\alpha }$ is the distance between the residue pair *i*,*j* Cα in the α fold (accession code 2OUG), $\sigma_{ij}^{\beta }$ is the distance between the residue pair *i*,*j* Cα in the β fold (accession code 2LCL), ε_C_ is the energy of the native contacts in the α and β folds, respectively, and $\varepsilon_{\text{C}}^{\text{IF}}$ is the energy of the interfacial contacts formed between the NTD and CTD of RfaH in the α fold (accession code 2OUG). In our simulations, the energy of native contacts in the α and β folds were equally weighted (ε_C_ = *k*_B_T* = ε), while the energy of the interdomain contacts $\varepsilon_{\text{C}}^{\text{IF}}$ was varied in the range {0,ε} to investigate the interplay between binding interface contacts and folding. The chosen sequence separation of two residues was adopted instead of the typical contact map definition of *i > j* + 3 due to two observations: i) simulations using the latter sequence separation gave rise to the presence of the intermediate I_2_ even when the strength of the interdomain contacts equaled ε (S7 Fig), while relaxation rates derived from NMR experiments on the wild-type protein demonstrated tight domain interactions and preservation in solution of the inactive structure of RfaH solved by crystallography [18]; ii) decreasing the strength of interdomain contacts on the dual-funneled model with a sequence separation of at least 3 residues significantly increased the population of the intermediates states, being higher than 70% when equilibrium between the α and β folds was achieved ($\varepsilon_{\text{C}}^{\text{IF}}=0.70\varepsilon$, S7 Fig), but there are no detectable intermediate configurations based on the signal from NMR experiments using the E48S mutant that reaches 1:1 equilibrium between both CTD folds [18]. Therefore, the dual funneled model developed herein is in better agreement with the available experimental evidence regarding the stability and conformational switching of RfaH in solution.

Because the structure of the NTD is well-conserved in all NusG family members [15] irrespective of the topology of the CTD, we did not allow the NTD to undergo unfolding, by treating all of its native contacts (obtained from the structure of the full RfaH protein in the α fold) with harmonic potentials instead of Lennard-Jones interactions. Also, dihedrals involving the modeled loop (residues 101–114) were disregarded. In total, 106 contacts from the α fold, 166 contacts from the β fold and 80 interfacial contacts between the RfaH NTD and CTD were included in the final model. Of these contacts, only 19 contacts are shared between the α and β folds of the CTD and were counted only once and given the native distance of the CTD in the α fold. This choice of contact distance was made such that the separation between the α and β folds in terms of the number of native contacts formed upon reaching each native basin was maximized, as shown in S8 Fig. Lastly, angle and dihedral contributions from both folds were included in the final model.

$$\begin{array}{c} V_{\text{angles}}^{db}=\sum_{\text{angles }\in \;\alpha }^{}\varepsilon_{\theta }{\left(\theta -\theta_{0}^{\alpha }\right)}^{2}+\sum_{\text{angles }\in \;\beta }^{}\varepsilon_{\theta }{\left(\theta -\theta_{0}^{\beta }\right)}^{2}\\ V_{\text{dihedrals}}^{db}=\sum_{\text{dihedrals }\in \;\alpha }^{}\varepsilon_{\phi }F_{D}\left(\phi -\phi_{0}^{\alpha }\right)+\sum_{\text{dihedrals }\in \;\beta }^{}\varepsilon_{\phi }F_{D}\left(\phi -\phi_{0}^{\beta }\right) \end{array}$$ (4)

This way of adding harmonic angle potentials, $V_{\text{angles}}^{db}$, has the effect of shifting the harmonic minimum to the average between the angles in the two structures, i.e. $\theta_{0}^{\alpha \beta }=(\theta_{0}^{\alpha }+\theta_{0}^{\beta })/2$. This symmetric potential homogeneously destabilizes both native structures and, thus, may lead to a reduced free energy barrier connecting the transition. However, the fact that removing this native basin destabilization from $V_{\text{dihedrals}}^{db}$ actually stabilizes the intermediate shows that these effects are difficult to predict (see S3 Fig). As is the case for the dihedrals, we do not expect the precise method used to mix the angles to affect the structural features of the TSEs or intermediate.

Simulations of the full RfaH protein using single and dual-basin potentials were performed in either an unmodified version of GROMACS 4.5.4 [48] or an in-house modified version that include a dual-basin dihedral potential [14]. For these structure-based models, reduced units are used. The timestep τ was 0.0005 and the temperatures ranged between 0.42 and 1.63 Ť, where the reduced temperature Ť = T/T* with *k*_B_T* = ε, where ε is the reduced energy unit. Since a functional RfaH seems to require a folded β domain in order to bind the ribosome, the temperature for analysis was calibrated to be just below folding temperature of β at $\varepsilon_{\text{C}}^{\text{IF}}=0$. All analysis is performed at T = 0.92T_F_^β^, where T_F_^β^ = 0.69 Ť and T_F_^β^ means the folding temperature of β. For each simulation, the structure-based models were equilibrated at each temperature for 5 × 10^6^ steps. Then, production runs using the replica exchange method [49] were performed for 5 × 10^8^ steps, allowing exchange between replicas every 10000 steps. For simulations where RNAp β’CC was explicitly included, the intramolecular native contacts of this segment where also treated with harmonic potentials. β’CC binding was considered as effective when the number of intermolecular contacts formed with RfaH NTD was higher than 60%. Thermodynamic parameters were computed as a function of the fraction of native contacts formed (Q, computed as in [5]) or as a function of the root mean square deviation (RMSD) from the solved structures. Multiple temperatures were combined using the weighted histogram analysis method (WHAM)[50]. For RfaH at $\varepsilon_{\text{C}}^{\text{IF}}=0.51\varepsilon$, one long constant temperature simulation of 5 × 10^8^ steps at T = 0.92 T_F_^β^ was also performed.

### Targeted molecular dynamics (TMD)

TMD simulations [42] allow driving of a subset of atoms to a target conformation by applying a steering force along an RMSD-based reaction coordinate. The functional form of the potential is:

$$U_{TMD}(t)=\frac{k}{2N}{\left(\text{RMSD}(t)-{\text{RMSD}}^{\text{*}}(t)\right)}^{2}$$ (5)

Where *k* is the spring constant, *N* is the number of atoms being steered, RMSD(*t*) is the RMSD between the current ensemble and the target conformation and RMSD*(*t*) is the linear decrease from the RMSD value between the initial structure and the target conformation to zero. For the TMD simulations, the full RfaH protein in the inactive α fold was solvated with TIP3P water molecules inside a box of 88 × 88 × 82 Å^3^ and ions were added to neutralize the net charge of the protein. The resulting system comprised 17,386 water molecules, 2,609 protein atoms and 2 chloride ions, and was treated using the Particle mesh Ewald method [51] and a non-bonded cut-off distance of 12 Å. The system was first minimized through 3 × 10^4^ iterations of the conjugated gradient algorithm, and then equilibrated for 5 ns at a constant temperature of 310 K, with a damping coefficient of 1 ps^-1^ for Langevin temperature control, and at a constant pressure of 1 atm, with a compressibility of 4.57 × 10^−5^ bar and a relaxation time of 100 fs for Berendsen pressure bath coupling, using an integration time step of 2 fs. After equilibration, TMD simulations were carried on for 20 ns, using an integration time step of 2 fs and a spring constant *k* = 672 kcal·mol^-1^·Å^-2^ over the 62 Cα atoms comprising the linker and CTD of RfaH (residues 101–162). Each TMD simulation was started from a configuration obtained through independent minimization and equilibration steps. All TMD simulations were performed using NAMD 2.9 [52] along with the AMBER ff99SB-ILDN force field [53], using periodic boundary conditions and SHAKE constraints. Secondary structure content was calculated using the Timeline extension of VMD [54].

### Local frustration and secondary structure propensity

Local frustration for RfaH CTD in the β fold (accession code 2LCL) and full RfaH in the α fold (accession code 2OUG) was calculated using the protein frustratometer [37] webserver. Analysis of local frustration addresses whether a given pair of residues in contact in the native state supports (minimal frustration, positive values) or conflicts (high frustration, negative values) with robust folding, compared to the same interaction being established in a different structural context (configurational frustration) or by different residues (mutational frustration).

Secondary structure propensity was estimated based on sequence analysis using the Jpred-3 [38] webserver, using neural network structure prediction (JNET), profile hidden Markov models (JHMM) and position-specific scoring matrix (JPSSM) methods.

## Supporting Information

S1 FigThermodynamic equilibrium of dual-funneled simulations of RfaH.The change in RMSD for both the α (A) and β folds (B) as a function of time and the exchange between replicas for 4 different replicas (C) for the dual-funneled model with $\varepsilon_{\text{C}}^{\text{IF}}=0.51\varepsilon$ is shown. To demonstrate sufficient sampling of the configurational space, two free energy landscapes calculated after splitting the resulting data from the replica exchange simulations in two halves are shown (D).(PDF)

S2 Fig Free energy landscapes of Rfah for different strengths of interdomain contacts. The strength of interdomain contacts was varied in the range {0,ɛ}. The free energy landscape of the unfolded state of RfaH (obtained at T = 1.81 T_F_^β^) is shown for comparison.
(PDF)

S3 FigFree energy landscapes of RfaH using mixed dihedrals and dual-basin dihedrals.Both simulations were done using the same strength for interdomain contacts $\left(\varepsilon_{\text{C}}^{\text{IF}}=0.51\varepsilon \right)$. The use of dual-basin potentials for the dihedral terms further stabilizes the intermediate configurations.(PDF)

S4 FigKinetic transitions between different ensembles during the reversible conformational change of RfaH.Counting of the number of transitions between the different states of the three-state folding mechanism of RfaH extracted from long constant temperature runs using the dual-basin model of RfaH with $\varepsilon_{\text{C}}^{\text{IF}}=0.51\varepsilon$.(PDF)

S5 Fig Local frustration and secondary structure propensity analysis of RfaH CTD. The mutational (residue identity) and configurational frustration (structural environment) of the native contacts of RfaH CTD in both folds is shown in A, with the color gradient indicating minimally frustrated contacts in green and highly frustrated contacts in red. The secondary structure propensity of RfaH CTD calculated using three different methods is shown in B, where the letter code is E for extended structures and H for helices.
(PDF)

S6 Fig Folding thermodynamics of RfaH upon deletion of RNAp-occluded interdomain contacts. The estimated free energy landscape shows that specific removal of the 53 NTD—CTD RfaH contacts, which would be occluded when RNAp β’CC binds to the NTD, leads to stabilization of the β fold.
(PDF)

S7 FigChoice of sequence separation for generation of the contact maps of RfaH.The free energy landscape of RfaH using a sequence separation *i* > *j* + 3 with $\varepsilon_{\text{C}}^{\text{IF}}=\varepsilon$ (A) and $\varepsilon_{\text{C}}^{\text{IF}}=0.70\varepsilon$ (B) and the estimated populations of each observed state for RfaH (C and D) shows that intermediate states are present even when the strength of interdomain interactions equals the strength of intradomain contacts and that their abundance is much higher than the native states when equilibrium between folds is achieved.(PDF)

S8 Fig Choice of contact distance of the 19 shared interactions between the α and β folds of RfaH CTD. The contact distance for interactions shared between folds was chosen such that the formation of native contacts for each basin was maximized.
(PDF)
