# Supplementary figures and images for: Interdomain Contacts Control Native State Switching of RfaH on a Dual-Funneled Landscape
Source: PLoS Comput Biol. 2015 Jul 31;11(7):e1004379. doi: 10.1371/journal.pcbi.1004379 (PMC4521827; doi:10.1371/journal.pcbi.1004379)

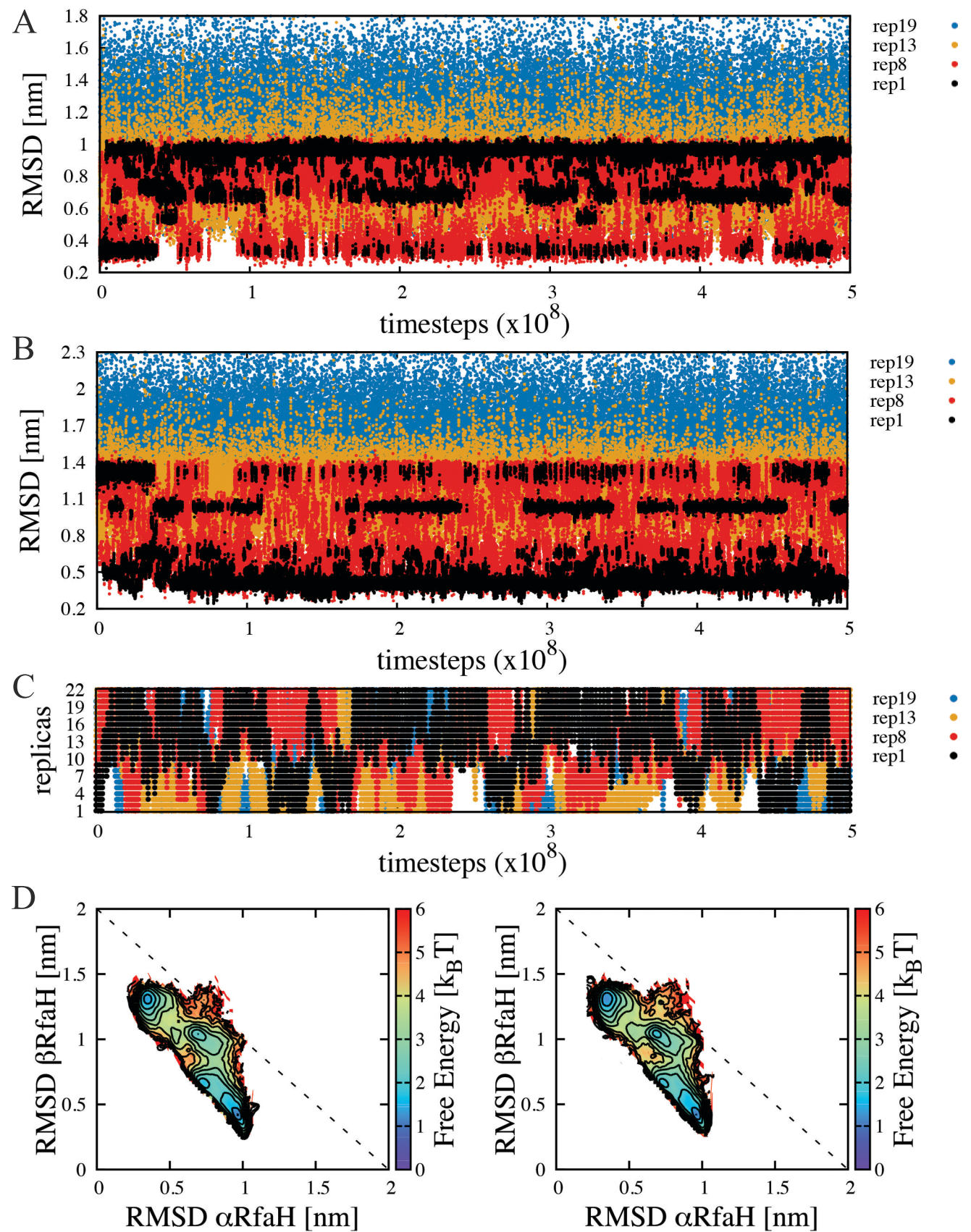

Supplement: S1 Fig — The change in RMSD for both the α (A) and β folds (B) as a function of time and the exchange between replicas for 4 different replicas (C) for the dual-funneled model with εCIF=0.51ε is shown. To demonstrate sufficient sampling of the configurational space, two free energy landscapes calculated after splitting the resulting data from the replica exchange simulations in two halves are shown (D). (PDF) [file pcbi.1004379.s001.pdf]

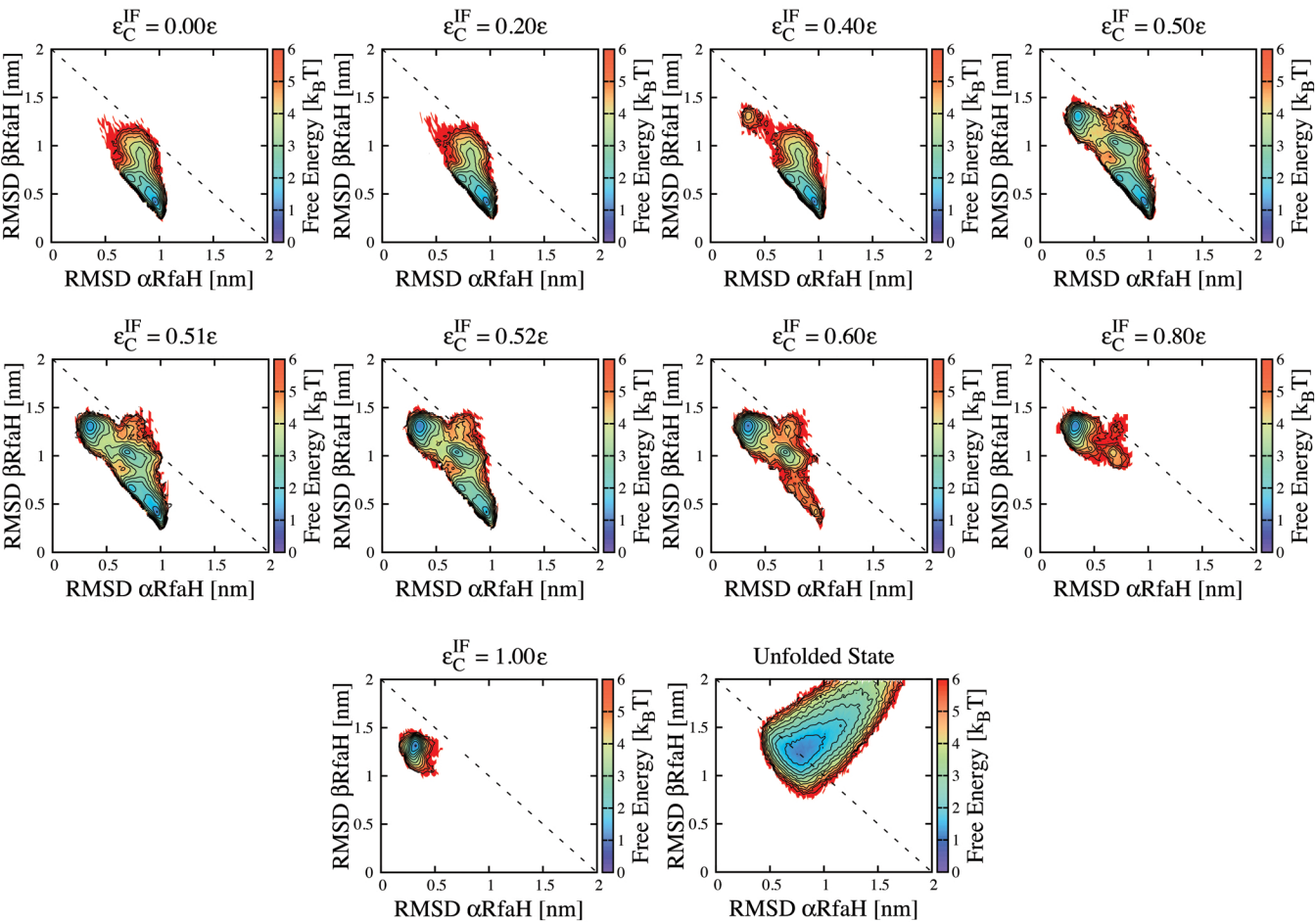

Supplement: S2 Fig — The strength of interdomain contacts was varied in the range {0,ɛ}. The free energy landscape of the unfolded state of RfaH (obtained at T = 1.81 TF β) is shown for comparison. (PDF) [file pcbi.1004379.s002.pdf]

mixed dihedrals

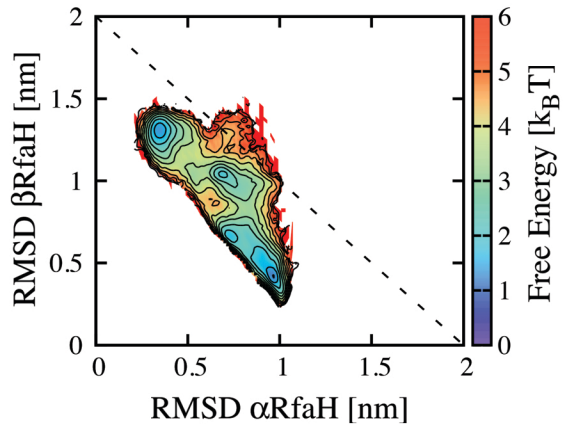

dual-basin dihedrals

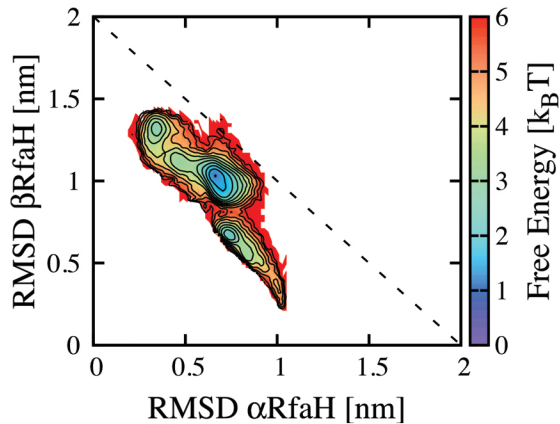

Supplement: S3 Fig — Both simulations were done using the same strength for interdomain contacts (εCIF=0.51ε). The use of dual-basin potentials for the dihedral terms further stabilizes the intermediate configurations. (PDF) [file pcbi.1004379.s003.pdf]

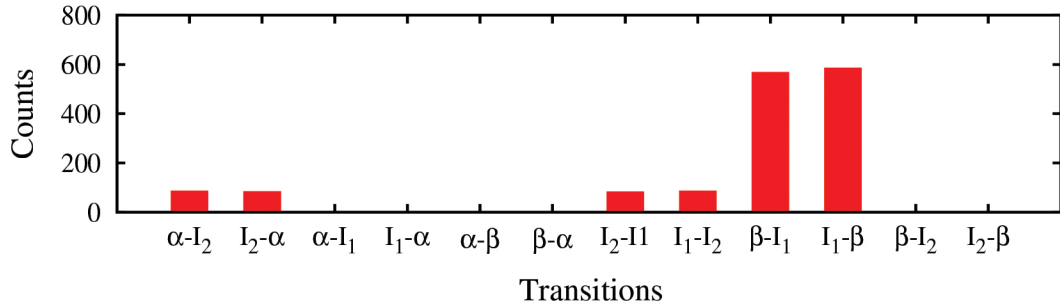

Supplement: S4 Fig — Counting of the number of transitions between the different states of the three-state folding mechanism of RfaH extracted from long constant temperature runs using the dual-basin model of RfaH with εCIF=0.51ε. (PDF) [file pcbi.1004379.s004.pdf]

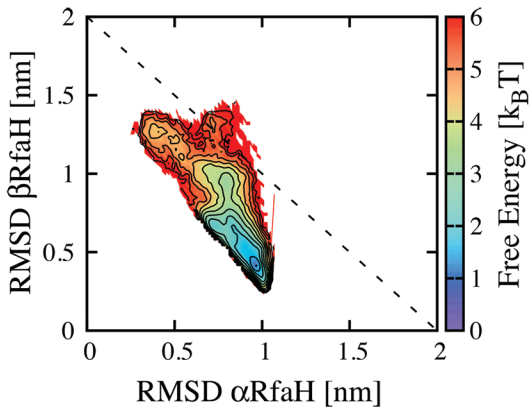

Supplement: S6 Fig — The estimated free energy landscape shows that specific removal of the 53 NTD—CTD RfaH contacts, which would be occluded when RNAp β’CC binds to the NTD, leads to stabilization of the β fold. (PDF) [file pcbi.1004379.s006.pdf]

A

$$i > j + 3, \quad \epsilon_C^{\text{IF}} = 1.00$$

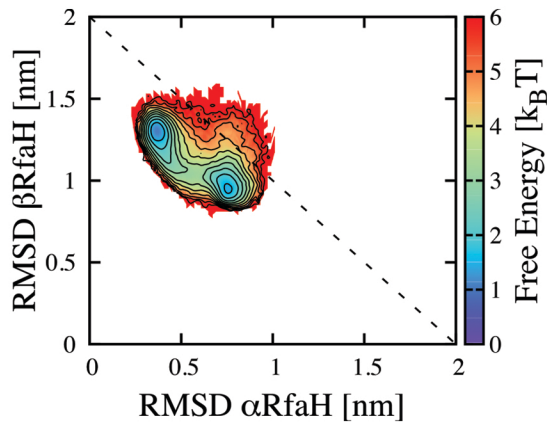

B

$$i > j + 3, \quad \epsilon_C^{\text{IF}} = 0.70$$

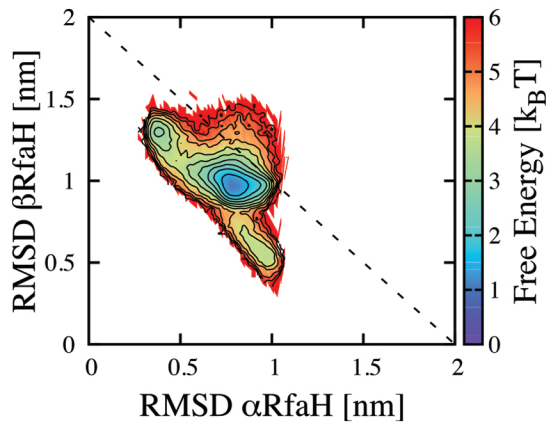

C

$$i > j + 2$$

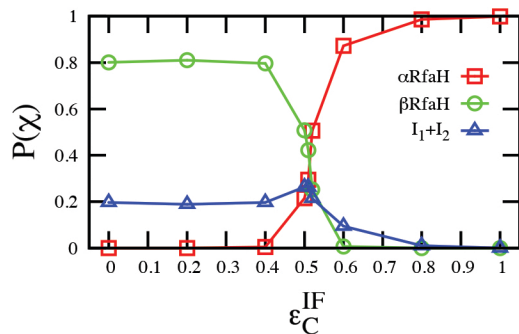

D

$$i > j + 3$$

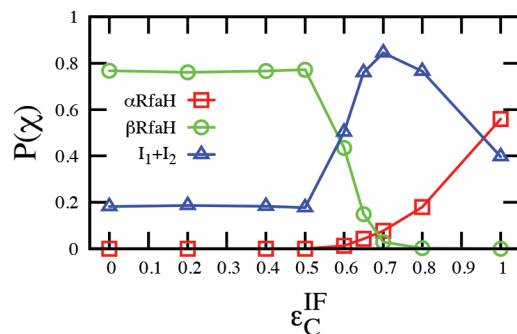

Supplement: S7 Fig — The free energy landscape of RfaH using a sequence separation i > j + 3 with εCIF=ε (A) and εCIF=0.70ε (B) and the estimated populations of each observed state for RfaH (C and D) shows that intermediate states are present even when the strength of interdomain interactions equals the strength of intradomain contacts and that their abundance is much higher than the native states when equilibrium between folds is achieved. (PDF) [file pcbi.1004379.s007.pdf]

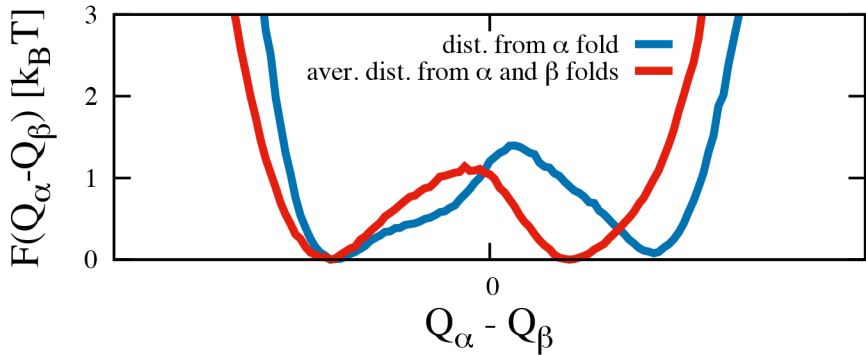

Supplement: S8 Fig — The contact distance for interactions shared between folds was chosen such that the formation of native contacts for each basin was maximized. (PDF) [file pcbi.1004379.s008.pdf]
