# Supplementary material for: Interdomain Contacts Control Native State Switching of RfaH on a Dual-Funneled Landscape
Source: PLoS Comput Biol. 2015 Jul 31;11(7):e1004379. doi: 10.1371/journal.pcbi.1004379 (PMC4521827; doi:10.1371/journal.pcbi.1004379)
Supplement: S5 Fig — The mutational (residue identity) and configurational frustration (structural environment) of the native contacts of RfaH CTD in both folds is shown in A, with the color gradient indicating minimally frustrated contacts in green and highly frustrated contacts in red. The secondary structure propensity of RfaH CTD calculated using three different methods is shown in B, where the letter code is E for extended structures and H for helices. (PDF) [file pcbi.1004379.s005.pdf]

A

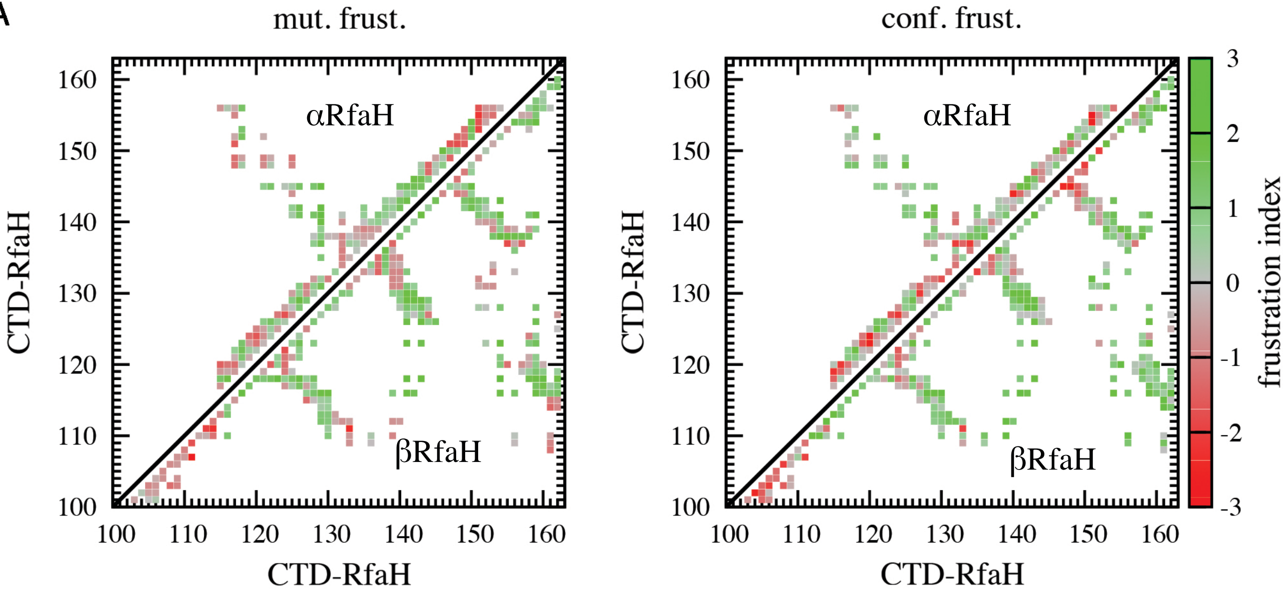

B

```
                 101-------111-------121-------131-------141-------151-------161-
RfaH-CTD         PKDIVDPATPYPGDKVIITEGAFEGFQAIFTEPDGEARSMLLLNLINKEIKHSVKNTEFRKL
 JNET            --------------EEEEE-------EEEEE------HEEEEHHH----EEEEEE-------
 JHMM            --------------EEEEE-------EEEEEE-----EEEEEEE-----EEEEE--------
 JPSSM           --------------EEEEE--------HHEE----HHHHHHHHHH----EEEEEE-------
```
